# A multi-modal treatment approach for the shoulder: A 4 patient case series

**DOI:** 10.1186/1746-1340-13-20

**Published:** 2005-09-16

**Authors:** Mario Pribicevic, Henry Pollard

**Affiliations:** 1 Macquarie Injury Management Group Department of Health and Chiropractic Macquarie University, 2109, Sydney Australia; 2 Macquarie Injury Management Group Department of Health and Chiropractic Macquarie University, 2109, Sydney Australia

**Keywords:** Shoulder, Impingement Syndrome, Multi-modal Treatment, Chiropractic

## Abstract

**Background:**

This paper describes the clinical management of four cases of shoulder impingement syndrome using a conservative multimodal treatment approach.

**Clinical Features:**

Four patients presented to a chiropractic clinic with chronic shoulder pain, tenderness in the shoulder region and a limited range of motion with pain and catching. After physical and orthopaedic examination a clinical diagnosis of shoulder impingement syndrome was reached. The four patients were admitted to a multi-modal treatment protocol including soft tissue therapy (ischaemic pressure and cross-friction massage), 7 minutes of phonophoresis (driving of medication into tissue with ultrasound) with 1% cortisone cream, diversified spinal and peripheral joint manipulation and rotator cuff and shoulder girdle muscle exercises. The outcome measures for the study were subjective/objective visual analogue pain scales (VAS), range of motion (goniometer) and return to normal daily, work and sporting activities. All four subjects at the end of the treatment protocol were symptom free with all outcome measures being normal. At 1 month follow up all patients continued to be symptom free with full range of motion and complete return to normal daily activities.

**Conclusion:**

This case series demonstrates the potential benefit of a multimodal chiropractic protocol in resolving symptoms associated with a suspected clinical diagnosis of shoulder impingement syndrome.

## Background

Practitioners of manual therapy commonly encounter patients presenting with shoulder pain and symptoms associated with rotator cuff pathology. Shoulder pain is the most common extraspinal complaint encountered in primary care clinics, and in clinical frequency is exceeded only by low back and neck pain [1]. Many shoulder conditions are associated with dysfunction of the rotator cuff [2-4].

Rotator cuff disorders represent a complex clinical entity requiring a thorough understanding and knowledge of shoulder anatomy, biomechanics and the functional relationship of the shoulder to nearby spinal structures including the cervical and thoracic spines.

Rotator cuff disorders commonly occur secondary to repetitive overuse (occupational or overhead throwing sports), which contributes to micro traumatic changes within rotator cuff tissue [5]. In addition, a single macro traumatic episode (fall on outstretched hand) can cause injury to rotator cuff tissue [5]. The normal aging process will also negatively influence the rotator cuff mechanism [2].

The most common source of shoulder pain originates from the rotator cuff tendons, with the most prevalent clinical diagnosis being impingement syndrome of the supraspinatus tendon [2-4,6].

Before discussing our case series it is important to review some important elements of taking a history and performing a shoulder physical examination. Certain clinical features may alert the practitioner to potentially serious causes (red flags) of shoulder pain, which constitute possible contra-indication to manual therapy [7,8] (Table 1). Other (yellow flag) features of the clinical history may affect the outcome of manual therapy and therefore recovery [7,8] (Table 2). A differential diagnosis list for shoulder pain [9] is seen in Table 3.

**Table 1:** Alerting features of a possible serious condition (red flag), which may present with shoulder pain [7,8].

***POSSIBLE SERIOUS CAUSES OF SHOULDER PAIN (RED FLAGS)***	
Signs of infection (fever)	Violent trauma
History of drug abuse	Swelling
Weight loss	Pain at rest
Age over 50	Night sweats
History of previous malignancy	History of fall
Constant, non mechanical pain	No precipitating event (for onset)
Palpable deformities of bone/tissue	HIV
Widespread neurological symptoms/signs	

**Table 2:** Possible features that may affect manual therapy outcome and ultimate patient recovery for patients presenting with shoulder pain (yellow flags) [7,8].

***YELLOW FLAGS***
Previous history of shoulder pain
Personal problems (alcohol, financial, marital)
Compensable injury
Unrealistic expectation of therapy
Long term absence from sport work
Belief that shoulder pain is dangerous
Dissatisfaction

**Table 3:** Describes the differential diagnosis for shoulder pain [9].

Referred pain from musculoskeletal sources	Cervical facet joints
	Thoracic facet joints
	Myofascial pain syndromes
Referred pain from visceral sources	Lungs
	Gallbladder
	Heart
	Diaphragm
Neuropathies	Brachial plexus neuropathies
	Peripheral neuropathies
Radicular pain	Cervical nerve root compression

Table 4[9] shows sources of shoulder pain mostly derived from local structures within the shoulder, whether due to trauma, overuse, arthritides or disease.

**Table 4:** Describes the sources of shoulder pain derived from local structures [9].

Trauma	Fracture
	Dislocation
	Tendon rupture
Overuse	Inflammation (tendinitis, bursitis)
	Capsular sprains
Arthritides	Osteoarthritis
	Rheumatoid variants
Other	Infection
	Neoplasm

This paper will discuss a common cause of shoulder pain and its largely unreported multi-modal conservative management in a chiropractic setting. This management will include pertinent aspects of the patient history, physical examination, differential diagnosis for shoulder pain as well as its management in 4 cases.

## Case Presentations

### Four presentations

A case of shoulder pain in a fit 42-year-old Caucasian male is presented. The pain was located diffusely in the postero-lateral aspect of the right shoulder and started gradually 4–6 weeks prior to presentation. No causative event was reported, although workplace activities required the patient to repetitively lift files above the shoulder level onto a shelf. Of note was the mention of a particularly busy period (increased intensity and duration) at work prior to the onset of pain.

The patient described the pain as being of a constant nagging and aching sensation with an intensity of 3/10 on the visual analogue scale (VAS). He also reported an intermittent sharp and catching sensation in the same location on shoulder abduction, with an intensity of 6/10 (VAS scale). No referred pain, or other neurological symptoms were reported, although he did report subjective weakness of the shoulder during elevation above shoulder level and inability to use the right arm comfortably.

Holding his arm on top of the steering wheel aggravated the pain when driving, as did sleeping on his right side, and also combing his hair. He described that heat packs provided short-term relief of pain. The patient reported no prior shoulder problems, no use of medication, and his medical, family and social history were otherwise unremarkable.

Physical examination of the right arm produced pain and restriction of movement at 50 degrees of right external rotation in the neutral position, with restriction and pain at 90 degrees of abduction. Both movements were guarded. An impingement sign was present, as confirmed by a positive Hawkins test. Hawkins test involves positioning the arm at 90 degrees of flexion with subsequent internal rotation. In addition Neers impingement test gave slight discomfort. Neer's impingement test is performed with the patient sitting as the practitioner stands behind the patient with one hand supporting the scapula to prevent scapula rotation and the other hand holding the forearm. The shoulder is brought into maximum flexion with a small degree of internal rotation. The test is considered positive if there is pain in the last 10–15 degrees of flexion. Pain is produced because the greater tuberosity is compressed against the anterior acromion or coracoacromial ligament, hence this test may aggravate an inflamed bursa (subacromial), the supraspinatus tendon or the anterior structures of the coracoacromial arch [10].

Muscle testing revealed slight weakness of the right infraspinatus muscle (Grade lV of V) and also right latissimus dorsi. Other routine shoulder tests revealed no abnormal findings (including instability testing, glenoid labrum testing, lateral slide test and muscle tests).

On palpation muscle spasm was noted in the right infraspinatus muscle and to a lesser extent the right rhomboid, supraspinatus and upper trapezius when compared to the other side. Significant focal tenderness was palpated over the rotator cuff insertion on the greater tuberosity of the humerus. Specific joint motion palpation revealed likely lateral flexion restriction of the right C5/6 lower cervical facet joint and left T2/3 thoracic facet joint with immobility of the right acromio-clavicular joint in an inferior direction.

The patient presented with X-rays, revealing no abnormalities.

A likely working diagnosis of a Primary Grade 2 Posterolateral Rotator Cuff Impingement (Neer classification-Table 5[11]) was determined.

**Table 5:** Neer classification of impingement [11].

STAGE l	Involving oedema and haemorrhage
STAGE ll	Involving fibrosis and tendonitis
STAGE lll	Involving degeneration (bone spurs) and tendon rupture

A second patient presenting was a slightly overweight 32 years old caucasian female with right-sided shoulder pain located superior, and in the postero-lateral aspect of the shoulder. The pain started 2 weeks prior to presentation after practising certain manual therapy manoeuvres of the lumbar spine at university. The patient was practising lumbar spine and sacro-iliac pisiform contact posterior-anterior manipulation. During this the shoulder is placed repetitively in a combined position of adduction, flexion and internal rotation. The patient described the pain as being a sharp, shooting sensation, intermittent, dependent on motion, with an intensity of 7/10 (VAS scale).

A diffuse aching sensation was also reported in the right upper deltoid region (so-called "military badge"). The pain was aggravated by elevation of the arm and sleeping on the right side. Relief was obtained by applying ice and taking anti-inflammatory/analgesic medication (Ibuprofen). The patient reported no prior shoulder problems, no general use of medication; her medical, family and social history were otherwise unremarkable.

Physical examination of the right shoulder revealed slight postero-lateral pain in the shoulder on external rotation and abduction. External rotation was restricted at 60 degrees and abduction at 90 degrees. Impingement was elicited with the Hawkins test and with the Neer's test. Other routine shoulder tests revealed no abnormal findings.

On palpation muscle spasm was notionally present in the right rhomboid major, upper trapezius, supraspinatus and particularly the infraspinatus. Trigger points were noted in the infraspinatus muscle with reproduction of the upper arm pain upon specific pressure. Motion palpation revealed likely right acromio-clavicular and sternoclavicular joint fixation, left T3/4 and right C5/6 lateral flexion restriction. The patient presented with plain film radiographs, which revealed no abnormality.

A likely working diagnosis of Grade 2 Primary Impingement of the rotator cuff (Neer classification-Table 5[11]) was determined. The working diagnosis also included the presence of an active infraspinatus myofascial pain syndrome.

The third patient was a slightly apprehensive 29-year-old Caucasian male with right-sided diffuse anterior and superior shoulder pain. The pain started gradually over an 8–10 week period, with the intensity being most prevalent during the 2 weeks prior to presentation. The patient was employed as a factory worker; a job that required combined repetitive shoulder movements and periods of administrative keyboard work.

The pain was described as a constant, deep, dull and nagging ache with an intensity of 5/10 (VAS scale). No neurological symptoms were reported, there were no dermatomal/sclerotomal pain referral patterns, although a slight diffuse aching sensation was mentioned in the right elbow and more prominently right "military badge" area. Together with the shoulder pain the patient reported a less intense (4/10) dull sensation specifically at the base of the cervical spine on the right and a vague headache like sensation at the base of the skull.

The right shoulder felt subjectively weaker with inability to lift the arm above shoulder level without pain. The pain was aggravated by specific arm postures and lying on the right side. There was no pertinent medical/family/social history.

Examination revealed a painful arc with onset of pain at 70 degrees abduction, external rotation being restricted at 70 degrees with a catching sensation at the end of motion. Reproduction of the pain was elicited with a Hawkins test and on supraspinatus muscle testing ("Empty can" test) revealing a grade 4 weakness and pain. Other routine shoulder tests revealed no abnormal findings.

Right cervical rotation restriction (65 degrees) was noted on the right, with a right Kemps joint stress test (combined right cervical rotation, lateral flexion and extension) reproducing the low cervical pain but no shoulder pain.

Palpation revealed muscle tenderness in the right supraspinatus, upper trapezius, levator scapulae and infraspinatus muscle groups. A trigger point was palpated in the infraspinatus muscle, which upon applying pressure reproduced the right upper arm diffuse ache. Palpating the rotator cuff insertion on the humerus and coracoacromial ligament caused significant tenderness. Motion palpation revealed likely joint restriction at the right C5/6 cervical facet joint, T2/3 and acromio-clavicular joint. Of interest was the postural presentation of a "rounded shoulder" and increased thoracic kyphosis.

A likely primary working diagnosis of a Grade ll Primary Rotator Cuff Impingement (Neer classification-Table 5[11]) with Supraspinatus tendonosis was determined, with secondary involvement of the cervical and thoracic spines.

The fourth patient presenting was a 40-year-old Caucasian female. She presented with right-sided anterior shoulder pain, which was nagging, aching and accompanied by a catching sensation on specific movements. The aching pain was constant with an intensity of 6.5/10 (VAS scale), while the catching pain was slightly more intense at 8/10. No neurological sensations were reported. The patient reported a diffuse aching pain in the posterior aspect of the shoulder over the scapula. Nothing relieved the pain, while arm elevation, driving, prolonged sitting behind the computer and poor posture made the pain worse.

The pain started 4 days prior to presentation after spending most of the weekend cleaning the walls at home with a sponge prior to painting. The patient had not had this pain before although due to her work (accountant) she often complains of posterior shoulder tension. The patient had been treated previously for an unrelated complaint (right sided sacroiliac area pain). The medical, family and social histories were unremarkable.

The physical examination revealed restriction in external rotation (60 degrees), and abduction with pain/catching at 90 degrees. Internal rotation was also tight and sore especially with the Hawkins test. The impingement sign was present with reproduction of the anterior pain with a Hawkins and Neers test.

Scapula dysfunction was also noted with a positive right-sided lateral glide test. It should be noted that no major difference was seen with the lateral glide test on the previous 3 patients.

Of importance was the postural presentation of anteriorly rotated shoulders, increased thoracic kyphosis and forward head carriage. A scoliotic curve was also noted with an apex convex to the right in the mid thoracic region. Palpation revealed muscle spasm in the right posterior shoulder girdle muscles with increased muscular tension and sensitivity to palpation in the right supraspinatus and infraspinatus compared to the left. Infraspinatus palpation revealed local muscle spasm with a reproduction of the posterior ache on specific pressure. Increased tenderness was noted whilst palpating the coracoacromial ligament and supraspinatus insertion on the humerus. Specific joint motion palpation revealed likely restriction in the right C5/6 joint, T3/4 and acromioclavicular joint.

A likely working diagnosis of a Grade ll, Primary Shoulder Rotator Cuff Impingement (Neer classification- refer to Table 5[11]) was determined. Of note was the secondary contribution of the scapula to this process. The working diagnosis also included the presence an active infraspinatus myofascial pain syndrome.

### The interventions

The 4 patients were admitted to a multimodal treatment protocol, which included the following interventions: soft tissue therapy, ultrasound phonophoresis, manipulation and exercise.

All of the patients received soft tissue therapy that involved the application of ischaemic pressure to the supraspinatus and infraspinatus muscles, as well as the rhomboids, upper trapezius and levator scapulae. The application involved palpating the muscle bellies and applying a sustained pressure into areas of muscle spasm until a release of the barrier of resistance was felt. Release meaning the relaxation of the point of muscle spasm with a decrease in the sensitivity and muscle tone after re-palpating the area. The pressure was applied repetitively, using a myofascial T-bar (a plastic, T shaped hand held tool with a rubber tip attached to the end in contact with the skin). Care was taken not to cause increased discomfort to the patient (to the level of pain tolerance).

Longitudinal and transverse friction massage was applied to the posterior tenomuscular junction of the infraspinatus muscle, the coracoacromial ligament (postero-inferior aspect) and the insertion of the supraspinatus on the greater tuberosity of the humerus. The friction massage application was achieved by palpating the capsular or tendinous adhesions and frictioning over its surface with the practitioner's index finger. This was maintained until friction anaesthesia was achieved and the patient could not feel any discomfort. A new point was then chosen and the process repeated. Once again care was taken to not cause excessive discomfort to the patient. At the end of the treatment sessions ice application was advised at a frequency of three applications of 15 minutes with two 20-minute breaks.

Ultrasound phonophoresis was applied to the areas that previously underwent friction massage with a topical corticosteroid [1% sigmacort]. Ultrasound was applied with a continuous wave form for 7 minutes at a setting of 2.2 W/cm^2 ^to the rotator cuff insertion on the anterior-inferior aspect of the humerus and posterior inferior aspect of the acromioclavicular joint.

Peripheral thrust manual manipulation was applied to the glenohumeral joints in external rotation (progressive) and inferiorly to the acromioclavicular joint and anterior to posterior to the sternoclavicular joint in all of the patients where a likely motion restriction was detected.

Mechanically assisted manipulations were also used with the Activator 2 apparatus in humeral external rotation or inferior through the AC joint. This particular technique was chosen for one of the female patients (fourth patient as an alternative) who expressed concern with peripheral manual manipulation after the first treatment session as an alternative technique.

Diversified spinal manipulations were used to manipulate the thoracic and cervical spines at the level of T3/4 and C5/6. All patients were given a basic exercise program with initial emphasis on isometric strengthening of the supraspinatus and infraspinatus muscles. This was implemented once a reduction in pain and improved range of motion was noted at a frequency of 4 sets of 10 repetitions, 2–3 times per day. Theraband (extendable elastic) exercises were also implemented at the same frequency after the initial isometric strengthening period. This also included shoulder shrugs, wall push-ups and scapula retraction exercises.

Patient 1 was treated for a total of 5 visits, patient 2 was treated 4 times, patient 3 was treated 5 times, and patient 4 was treated 4 times.

At the end of the last treatment session (5 and 4 treatments respectively) a repeat physical examination revealed a full and painless range of motion with no subjective symptoms, and negative orthopaedic testing (Hawkin's and Neer's). Patient 1 was seen 4 weeks later for a new and unrelated complaint, who after questioning reported no shoulder complaints (pain). Full range of motion was maintained. Patient 2 was contacted via the phone and upon questioning also reported no subjective pain and full return to normal activities at 1 month post treatment. Patient 3 was followed at 4 and 8 weeks after the last treatment revealing no subjective and objective symptoms. Patient 4 was seen at 4 and 8 weeks with no symptoms of impingement reported and no objective findings.

## Discussion and Conclusions

Rotator cuff impingement and or tendonosis is a common disorder encountered in a primary health care setting [12-15]. Perhaps, less in chiropractic practises as opposed to medical and physiotherapy. To date, there are no data investigating the prevalence of shoulder pain in the chiropractic setting. This may be due to the lack of general public awareness about the scope and capabilities of chiropractors to be involved in management of non-spinal disorders or simply the public making another choice. This condition presents a challenge to the chiropractor due to its prevalence, and its possible close interrelationship with the spine.

A major reason for documenting this treatment protocol is to encourage the development of future clinical guidelines for chiropractors and to encourage the expansion of their treatment range to include peripheral disorders.

Another goal of this report is to highlight that multimodal management is often required to address the painful shoulder and not to determine or show which treatment approach or particular therapy was more effective. The four patients in this paper were managed with a treatment protocol that included a number of therapies. The literature [16-22] suggests that the multimodal approach is an appropriate method for the successful conservative management of shoulder problems.

The cervical and thoracic spines should be reviewed as a possible factor associated with rotator-cuff dysfunction. As an example consider the slumping posture in a competitive swimmer. Others and we hypothesise that the rounded shoulders and increased thoracic kyphosis places increased demands on the rotator cuff and contributes to the impingement process [23]. A possible mechanism for this hypothesis is as follows: the posture may alter the mechanical function (orientation) of the scapula and humerus, leading to muscular imbalances, abnormal movement patterns during glenohumeral elevation with associated weakness of the posterior cuff muscles. Therefore this may lead to a loss of force couple at the glenohumeral joint with resultant repetitive humeral head impingement [23-25].

The outcome measures for the study included improvement of pain, return to pre-treatment activities, and restoration of full active and passive movements. The outcome measures were mainly subjective in nature and dependent on the response of the patients and the practitioner's skill in conducting the orthopaedic reassessment, therefore allowing an element of examination bias. This particular shortcoming may be improved by using more sensitive scoring systems that can be accurately reproduced by different observers such as the subjective shoulder rating system [26], UCLA scoring system [27], or the highly sensitive Constant/Murley functional assessment of the shoulder [28].

Although frequently advocated for outcomes based assessment, goniometric measurement for the shoulder remains questionable. Williams et al [29] studied 22 observers who used three different types of goniometers to assess the range of abduction and visual estimation. The results demonstrated visual estimation to be the most reliable method. Moderate inter-observer reliability was also demonstrated in a study by Bostrom et al [30] where range of motion was measured using a goniometer.

This report presents an approach that combines aspects of traditional forms of chiropractic, physiotherapy and medicine in the conservative management of certain shoulder pain.

The individual therapies that were used in this multimodal treatment protocol have been shown to be useful in the management of shoulder pain both singularly and in combination [18,19,31-36].

Of the electro-modalities the apparatus used was ultrasound. Some authors routinely advocate the usage of ultrasound in conjunction with other modalities and report positive outcomes [3,16,35]. The physiologic benefits of ultrasound have been attributed to its thermal actions; these involve an increase in peripheral blood flow, increased tissue metabolism and greater tissue extensibility [37].

The use of ultrasound for shoulder pain and its effect on soft tissue structures of the shoulder has been studied extensively in the literature. A recent study by Nykanen [36] investigating pulsed ultrasound treatment of the painful shoulder in a randomised, double blind and placebo controlled study, showed no differences in outcome between the treatment and placebo groups at the end of the trial period. However, when the ultrasound was used to complement treatment the patients reported a significant subjective improvement in symptoms. A further study by Downing [35], and Perron et al [38], also showed no apparent benefit from ultrasound therapy. None of these studies demonstrated statistically significant results supporting ultrasound therapy. A recent review of the literature conducted by Van der Windt [39] also concluded that there is little evidence that ultrasound therapy is effective for soft tissue disorders of the shoulder. By contrast to the above studies the subjects in this paper were treated with a 3MHz setting plus phonophoresis that may have influenced the outcome measures. Nevertheless the efficacy and effectiveness of ultrasound for shoulder pain remains in doubt.

In this study the subjects were also treated with an ultrasound technique known as phonophoresis. Phonophoresis involves the movement of a medication through intact skin into the underlying soft tissue, by ultrasonic pertubation [37]. By using ultrasound a topical corticosteroid cream can be successfully delivered across the skin with a view to reducing the inflammation and pain associated with the more superficial soft tissue injuries and disorders [40]. Davick [40] showed in his study corticosteroid medication penetration through to the epidermal layer of skin, and further into the stratum corneum. The medication used to treat the subjects was a topical corticosteroid – Sigmacort 1%. This approach combined with the therapeutic effects of ultrasound appeared subjectively to have a beneficial effect as a treatment adjunct.

There is some evidence reporting the positive effects of phonophoresis. Griffin et al [41] conducted a double blind study comparing the effects of phonophoresis and ultrasound in 102 patients with various shoulder complaints. Of the subjects receiving phonophoresis 68% showed significant improvement in range of motion and pain as opposed to 28% in the ultrasound group.

In 1999 one paper by chiropractors investigated the benefits of phonophoresis. Gimblet et al [16], reported treating two subjects with calcific tendonitis by using soft tissue therapy, phonophoresis and manipulation. Both subjects at the end of the treatment protocol experienced complete resolution of symptoms.

Transverse friction massage has been advocated by a number of authors in the management of shoulder disorders [19,34]. Hammer describes friction massage as a technique where an involved muscle, tendon or ligament is massaged by applying pressure with a reinforced finger [19,34]. The transverse motion across the involved tissue and the resultant hyperaemia are said to be the chief healing factors of friction massage [19,34]. The transverse action is said to prevent the formation of scar tissue while longitudinal friction effects the transportation of blood and lymph [19].

The traumatic hyperaemia is postulated to release histamine and bradykinins resulting in vasodilation and reduction of oedema [34]. Friction massage is said to stimulate the proliferation of fibroblasts and collagen fibre realignment with cross linkages [39].

It is reported that up to two weeks are required for mature cross-links to form [24]. In the acute stage a light friction is suggested while in the chronic condition, a stronger pressure may be required [34]. Hammer [19] also describes the successful management of a chronic bursitis by the use of soft tissue friction massage.

The management of the subjects in this paper also included orthopaedic, motion assessment and treatment of spinal structures including the cervical and thoracic spines. Diversified spinal adjustments were directed at the identified hypo mobile motion segments of the cervical and thoracic spines. This included assessment and adjustment of the glenohumeral joint in restricted planes of motion.

It is postulated that abnormal thoracic and cervical spine postural alignment (with any associated spinal joint fixation) may alter the resting position of the scapula contributing to problems of the rotator cuff musculature [23]. In our cases changes in the lateral spinal curves were particularly noted in the third and fourth patients [23].

Abnormal spinal curves can result from chronic poor posture which may result in shoulder girdle muscle imbalance, altered muscle length tension relationships, joint incongruity, ligamentous laxity, changes in arthrokinematics and gross shoulder motion [23].

As noted by many clinicians a commonly related postural condition is that associated with anterior head carriage associated with rounded shoulders [19,23]. This type of postural deviation often causes a compensatory extension at the atlanto-occipital articulation, reversal or flattening of the cervical lordosis, thoracic kyphosis, protraction of the scapulae with the inferior angle of the scapula moving medially whilst the glenoid fossa moves anterior and inferior, and finally internal rotation of the humerus.

As a result, muscle imbalances of the shoulder girdle may occur. These potentially include parascapular muscle weakness, winging of the scapula, altered scapula position, and scapula dysrhythmia [10,23]. Also, weakness of the posterior rotator cuff muscles may influence the force couple mechanism at the glenohumeral joint causing a resultant upward shear of the humeral head during elevation of the arm.

**Figure 1:**
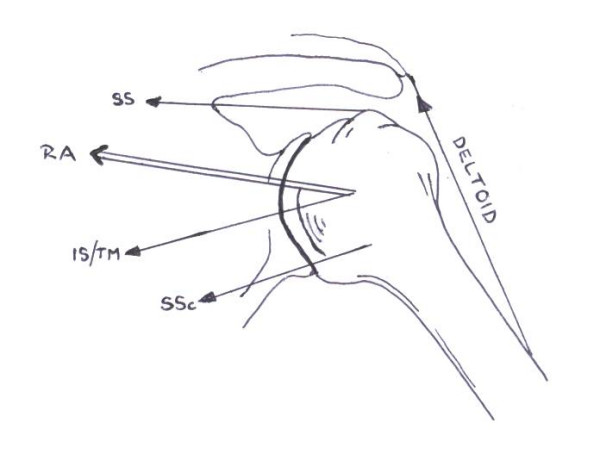
The glenohumeral force couple. The resultant force (action) of the rotator cuff muscles results in compression and inferior glide of the humeral head during elevation. (RA = resultant action, Deltoid, SS = Supraspinatus, SSc = Subscapularis, IS = Infraspinatus and TM = Teres Minor) [42].

During shoulder elevation the dominant force vector is provided by the deltoid muscle and in a superior direction. Under normal circumstances the cuff muscles will counter this superior shear in the opposite direction, creating a stabilizing and compressive action of the humeral head with respect to the glenoid during elevation. A diagrammatic representation of the gleno-humeral force couple [42] is seen in Figure 1. With cuff weakness (even slight) the force couple may be altered enabling an abnormal upward displacement of the humeral head and the impingement of the subacromial structures and the humeral head against the under surface of the acromion [10,23].

Repetition of this process may cause irritation of pain producing structures creating shoulder pain syndromes. In order to address the abnormal force couple and its potentially causative mechanism, specific exercises were introduced to help restore strength and muscular functioning of the glenohumeral joint and scapula articulations. (That is, once motion was normalised).

It is acknowledged that a significant weakness in this case series is the lack of imaging using diagnostic ultrasound to confirm the diagnosis of impingement or indeed some other cause for the pain. We encourage a further study of the treatment protocol described above. This study should be a randomised controlled trial and include diagnostic ultrasound confirmed impingement.

Successful management of rotator cuff impingement and related shoulder pain syndromes should include the consideration of potential sources of shoulder pain. Also the function of the implicated structures in global shoulder function should be reviewed. This should include the associated structures of the scapulohumeral, scapulothoracic articulations, the cervical and the thoracic spine.

This paper highlights a successful outcome for 4 subjects with clinically diagnosed shoulder impingement syndrome after receiving a multimodal treatment approach in a chiropractic setting.

## Authors' contributions

MP provided treatment to the subjects, participated in the design and helped draft the manuscript.

HP conceived of the study, participated in its design and helped to draft and edit the manuscript. All authors read and approved the manuscript.
